# Impact of BMI on peak growth hormone responses to provocative tests and therapeutic outcome in children with growth hormone deficiency

**DOI:** 10.1038/s41598-019-52644-1

**Published:** 2019-11-07

**Authors:** Aram Yang, Sung Yoon Cho, Min Jung Kwak, Su Jin Kim, Sung Won Park, Dong-Kyu Jin, Ji-Eun Lee

**Affiliations:** 1 Department of Pediatrics, Kangbuk Samsung Hospital, Sungkyunkwan University School of Medicine, Seoul, Republic of Korea; 2 Department of Pediatrics, Samsung Medical Center, Sungkyunkwan University School of Miedicine, Seoul, Republic of Korea; 3 Department of Pediatrics, Pusan National University Hospital, Pusan National University School of Medicine, Busan, Republic of Korea; 4 Department of Pediatrics, Inha University Hospital, Inha University College of Medicine, Incheon, Republic of Korea; 5 Department of Pediatrics, Gangseo MizMedi Hospital, Seoul, Republic of Korea

**Keywords:** Paediatric research, Endocrinology, Endocrinology, Paediatric research

## Abstract

This study investigated the relationship between peak stimulated growth hormone (GH) and body mass index (BMI), as well as the impact of BMI on therapeutic response in patients with GH deficiency (GHD). A total of 460 patients were enrolled in the study. The patients were divided into four groups as per the etiology and peak GH values: idiopathic (n = 439), organic (n = 21), complete (n = 114), and partial (n = 325) GHD groups. Subsequently, they were classified as normal, overweight, or obese based on their BMI. There was no difference in BMI between complete and partial GHD. A significant negative relationship between peak GH and BMI were found. Moreover, obese GHD children had a considerably better therapeutic response in height increase and BMI decrease during 2 years of GH treatment compared to non-obese children with GHD. There was no difference between peak GH and type of GH stimulation test (GHST), except the clonidine test, which showed a much lower peak GH in obese GHD children. In conclusion, BMI had a negative impact on peak GH response, and therapeutic outcome was more favorable in the obese group. Despite no difference in GH response by type of GHST, the degree of obesity differentially affected the results.

## Introduction

Children with growth hormone deficiency (GHD) have a significantly short stature for their age and gender and a deteriorated growth velocity^1^. GHD is diagnosed when at least two responses of GH stimulation tests (GHSTs) performed with various pharmacologic stimuli such as insulin, clonidine, glucagon, arginine, L-dopa, or growth hormone-releasing hormone (GHRH) are below normal^2,3^. However, GH secretion is affected by various factors including age, pubertal status, different pharmacological growth hormone stimuli, and body mass index (BMI)^4^. The interpretations of provocative tests also remain problematic for different test protocols and multiple types of assays by different laboratories^5,6^. Thus, these tests may show relatively lower sensitivity and specificity, and as such, careful analysis and test selection are required, taking into consideration various related factors.

Obesity is a well-known confounder of impaired spontaneous GH secretion and reduced GH responses to provocative tests^7,8^. As in adults, obesity in children is associated with reduced GH secretion which contributes to a shift towards visceral adiposity^9^ and an increase in cardiovascular risk markers^10^ which can be restored by weight loss or short-term caloric restriction^11,12^. The effects of obesity on GH levels have mainly been studied in adults with GHD or obesity, and in obese children or children with idiopathic short stature (ISS)^8,13–18^. However, although overdiagnosis of GHD can occur due to recent increases in overweight and obese children, few studies have been conducted in children with GHD^8,19,20^ and there is currently no available BMI-specific cutoff value for peak GH in children.

Thus, the aim of this study was to investigate the therapeutic response according to obesity status, and the relationship between GH secretion and BMI in children with GHD.

## Results

### Clinical characteristics of the study subjects

**Figure 1:**
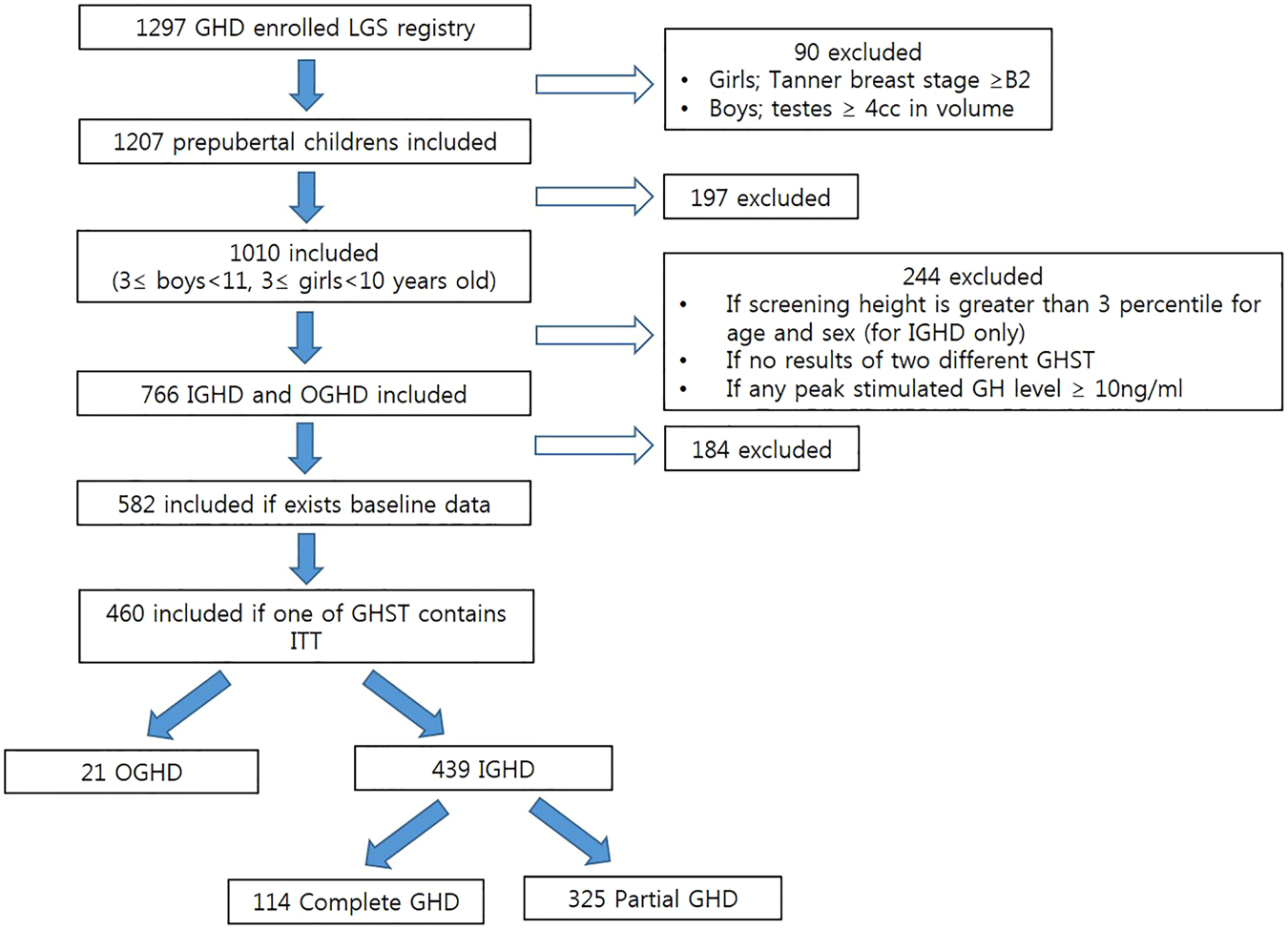
Schematic diagram of patient selection and classification. *GHD* growth hormone deficiency, *GHST* growth hormone stimulation test, *IGHD* idiopathic growth hormone deficiency, *ITT* insulin tolerance test, *OGHD* organic growth hormone deficiency.

A schematic diagram of the study is shown in Figure 1. Of the 460 patients with GHD, 439 had idiopathic GHD (IGHD), 21 had organic GHD (OGHD), 114 had complete GHD (CGHD), and 325 had partial GHD (PGHD). The baseline characteristics of each group stratified by obesity status are presented in Tables 1 and 2.

**Table 1:** Demographic characteristics of IGHD and OGHD.

	Idiopathic GHD				Organic GHD				IGHD	OGHD	*P* value
	Normal (n = 385)	Overweight (n = 28)	Obese (n = 26)	Normal/OW (n = 413)	Normal (n = 16)	Overweight (n = 3)	Obese (n = 2)	Normal/OW (n = 19)	Total (n = 439)	Total (n = 21)	
Gender (M/F)	146/239	16/12	12/14	162/251	3/13	2/1	1/1	5/14	174/265	6/15	NS
Age at GHT start (year)	7.21 (5.43, 9.64)	6.18 (5.33, 9.21)	6.53 (4.93, 8.58)	7.15 (5.43, 9.59)	8.57 (5.96, 9.56)	6.21 (5.45, 8.81)	6.68 (5.81, 7.55)	8 (5.45, 9.52)	7.1 (5.41, 9.56)	7.9 (5.81, 9.42)	NS
GH dose at GHT start	0.26 (0.22, 0.34)	0.24 (0.22, 0.32)	0.26 (0.19, 0.48)	0.26 (0.22, 0.34)	0.24 (0.21, 0.3)	0.24 (0.24, 0.27)	0.24 (0.18, 0.29)	0.24 (0.21, 0.29)	0.26 (0.22, 0.35)	0.24 (0.21, 0.29)	NS
Bone age (year)	5 (3.1, 7.5)	3.8 (3, 6.5)	3.75 (2.8, 6.5)	5 (3, 7.5)	5.85 (5, 9)	5.6 (5, 9)	3.5 (3.5, 3.5)	5.7 (5, 9)	5 (3.0, 7.5)	5.65 (5, 9)	NS
Height SDS	−2.68^a^ (−3.08, −2.42)	−2.8^a^ (−3.25, −2.35)	−2.43^a,b^ (−2.61, −2.28)	−2.68^b^ (−3.11, −2.42)	−2.87 (−3.15, −2.06)	−2.09 (−2.62, −1.76)	−0.51^b^ (−0.9, −0.12)	−2.62^b^ (−3.11, −1.88)	−2.65 (−3.08, −2.41)	−2.62 (−2.95, −1.76)	NS
Weight^a^ SDS	−2.07^a^ (−2.62, −1.49)	−0.65^a^ (−0.92, −0.42)	0.04^a,b^ (−0.22, 0.3)	−2.02^b^ (−2.58, −1.38)	−1.7^a^ (−2.31, −1.18)	−0.07^a^ (−0.64, 0.15)	1.75^a,b^ (1.62, 1.89)	−1.36^b^ (−2.11, −0.64)	−1.91 (−2.54, −1.26)	−1.28 (−1.93, −0.07)	0.02
BMI SDS	−0.55^a^ (−1.18, 0.07)	1.23^a^ (1.11, 1.33)	1.98^a,b^ (1.69, 2.17)	−0.45^a,b^ (−1.15, 0.23)	−0.07^a^ (−1.44, 0.23)	1.39^a^ (1.14, 1.46)	2.78^a,b^ (2.7, 2.85)	0.05 (−1.39, 0.48)	−0.38 (−1.12, 0.44)	0.16 (−0.24, 0.55)	NS
IGF-1 SDS	−0.9 (−1.35, −0.43)	−0.44 (−1.09, 0.06)	−0.78 (−1.1, −0.24)	−0.87 (−1.33, −0.41)	−0.96 (−2.13, 0.52)	−0.69 (−1.99, 0.6)	−1.58 (−1.87, −1.3)	−0.96 (−2, 0.6)	−0.87 (−1.32, −0.39)	−1.18 (−2.0, 0.2)	NS
IGFBP-3 SDS	−0.39 (−1.38, 0.86)	0.11 (−1.1, 1.08)	−0.4 (−1.22, 0.5)	−0.35 (−1.33, 0.98)	−1.15 (−2.69, −0.5)	−1.01 (−0.35, 0.33)	−0.75 (−1.08, −0.43)	−1.15 (−2.52, −0.08)	−0.37 (−1.31, 0.95)	−0.95 (−2.35, −0.43)	NS
Height − MPH SDS	−1.87 (−2.42, −1.44)	−1.68 (−3.24, −1.09)	−1.43 (−2.14, −1.23)	−1.86 (−2.44, −1.41)	−2.31 (−2.93, −1.47)	−1.97 (−2.91, −1.88)	−0.53 (−1.53, 0.48)	−2.15 (−2.91, −1.7)	−1.85 (−2.43, −1.39)	−2.11 (−2.91, −1.47)	NS
Duration of GHT (year)	3.59 (1.81, 4.93)	3.31 (1.58, 4.27)	3.74 (1.61, 4.85)	3.59 (1.78, 4.91)	5.72 (4.4, 7.2)	6.48 (3.72, 6.81)	7.0 (4.73, 9.28)	5.87 (3.72, 6.96)	3.6 (1.75, 4.9)	5.87 (4.73, 6.96)	<0.0001

Data are expressed as median (IQR).

^*^Significant association was classified as *P* < 0.05.

*BMI* body mass index, *GH* growth hormone, *GHD* growth hormone deficiency, *GHT* growth hormone treatment, *IGF-1* insulin-like growth factor-1, *IGFBP-3* insulin-like growth factor-binding protein 3, *IGHD* idiopathic growth hormone deficiency, *MPH* midparental height, *NS* not significant, *OGHD* organic growth hormone deficiency, *OW* overweight, *SDS* standard deviation score.

GH dose at GHT start (mg/kg/week).

^a^*P* < 0.05 for the difference of three groups normal, overweight, and obese (*P* value obtained from ANOVA test/ Kruskal-Wallis test).

^b^*P* < 0.05 for the difference between Obese *vs*. Normal/OW group (*P* value obtained from two-sample t-test/ Wilcoxon rank-sum test).

**Table 2:** Demographic characteristics of CGHD and PGHD.

	Complete GHD			Partial GHD			CGHD	PGHD	*P* value
	Obese (n = 9)	Normal/OW (n = 105)	*P* value	Obese (n = 17)	Normal/OW (n = 308)	*P* value	Total (n = 114)	Total (n = 325)	
Gender (M/F)	6/3	33/72	NS	6/11	129/179	NS	39/75	135/190	NS
Age at GHT start (year)	7.07 (6.09, 8.58)	7.59 (5.51, 9.92)	NS	6.06 (4.87, 8.55)	7.12 (5.41, 9.53)	NS	7.33 (5.51, 9.91)	7.02 (5.29, 9.51)	NS
GH dose at GHT start (mg/kg/week)	0.29 (0.23, 0.55)	0.26 (0.22, 0.38)	NS	0.2 (0.17, 0.29)	0.26 (0.22, 0.33)	NS	0.26 (0.22, 0.39)	0.25 (0.21, 0.33)	NS
Bone age (year)	5.65 (4, 6.5)	5 (3, 8)	NS	3.4 (2.8, 6.75)	5 (3.3, 7.5)	NS	5 (3, 8)	5 (3, 7.5)	NS
Height SDS	−2.52 (−2.64, −2.46)	−2.73 (−3.2, −2.44)	NS	−2.34 (−2.54, −2.27)	−2.67 (−3.09, −2.41)	0.001	−2.7 (−3.2, −2.44)	−2.63 (−3.06, −2.4)	NS
Weight SDS	0.04 (−0.42, 0.16)	−2.01 (−2.58, −1.28)	<0.0001	0.04 (−0.2, 0.35)	−2.02 (−2.58, −1.38)	<0.0001	−1.89 (−2.53, −1.05)	−1.97 (−2.54, −1.31)	NS
BMI SDS	1.88 (1.74, 2.06)	−0.54 (−1.12, 0.47)	<0.0001	1.98 (1.68, 2.19)	−0.42 (−1.17, 0.21)	<0.0001	−0.36 (−1.08, 0.7)	−0.38 (−1.13, 0.33)	NS
IGF−1 SDS	−0.92 (−1.42, 0.68)	−0.96 (−1.55, −0.51)	NS	−0.72 (−1.1, −0.35)	−0.82 (−1.29, −0.36)	NS	−0.95 (−1.51, −0.45)	−0.82 (−1.28, −0.36)	NS
IGFBP-3 SDS	−1.11								
(−1.87, −0.99)	−0.46 (−1.36, 0.92)	NS	−0.26 (−0.91, 2.07)	−0.32 (−1.32, 1.02)	NS	−0.56 (−1.36, 0.76)	−0.28 (−1.27, 1.04)	NS	
Height-MPH SDS	−1.41 (−3.26, −0.61)	−2 (−2.87, −1.51)	NS	−1.44 (−2.1, −1.32)	−1.78 (−2.35, −1.37)	NS	−1.97 (−−2.92, −1.43)	−1.77 (−2.33, −1.36)	0.04
Duration of GHT (year)	4.05 (3.1, 4.9)	3.53 (1.61, 4.92)	NS	3.72 (1.44, 4.62)	3.62 (1.81, 4.89)	NS	3.56 (1.64, 4.92)	3.63 (1.79, 4.87)	NS

Data are expressed as median (IQR).

^*^Significant association was classified as *P* < 0.05.

*BMI* body mass index, *CGHD* complete growth hormone deficiency, *GH* growth hormone, *GHD* growth hormone deficiency, *GHT* growth hormone treatment, *IGF-1* insulin-like growth factor-1, *IGFBP-3* insulin-like growth factor-binding protein 3, *MPH* midparental height, *NS* not significant, *OW* overweight, *PGHD* partial growth hormone deficiency, *SDS* standard deviation score.

### IGHD vs. OGHD

The median age of the IGHD group at GH start was 7.1 years, which was similar to that of OGHD. There was no significant difference between the IGHD and OGHD groups in terms of gender distribution, GH dose at GH treatment (GHT) initiation, bone age, height standard deviation score (SDS), BMI SDS, insulin growth factor-1 (IGF-1) SDS, IGF-binding protein-3 (IGFBP-3) SDS, and height–midparental height (MPH) SDS. In all, 47.6% of patients with OGHD had multiple pituitary hormone deficiency in conjunction with other pituitary deficiencies. Among them, levothyroxine (n = 10) was the most common hormone supplemented, followed by hydrocortisone (n = 6), desmopressin (n = 5), and sex-steroid hormone (n = 4). When classified by obesity status, the height SDS of obese patients in both the IGHD and OGHD groups was greater than that of the Normal/Overweight (OW) group (IGHD: −2.43 [Obese] *vs*. −2.68 SDS [Normal/OW], *P* = 0.002; OGHD: −0.51 [Obese] *vs*. −2.62 SDS [Normal/OW], *P* = 0.04). Baseline IGF-1 SDS did not significantly differ according to obesity status in the IGHD and OGHD groups. However, in the first year of GHT, IGF-1 SDS significantly increased in the Obese group and was significantly higher compared to the Normal/OW group (IGF-1 SDS at 1 y: 1.93 [Obese] *vs*. 0.49 [Normal/OW]; *P* < 0.0001; Supplementary Fig. S1). Baseline height–MPH SDS was similar between the IGHD and OGHD groups.

### CGHD vs. PGHD

The demographics of the CGHD and PGHD groups did not significantly differ except that the height SDS of the Obese group in PGHD was significantly higher than that in the Normal/OW group (−2.34 *vs*. −2.67 SDS, *P* = 0.001). Baseline height–MPH SDS was lower in the CGHD group than in the PGHD group (−1.97 *vs*. −1.77 SDS, *P* = 0.04).

### Peak-stimulated GH according to provocation test

**Figure 2:**
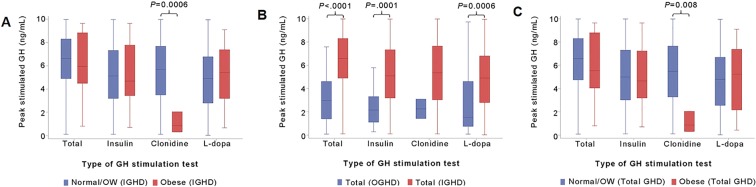
Peak-stimulated GH according to provocation tests for total GHD, IGHD, and OGHD. Data are expressed as the median (IQR). Box plot showing median level of peak-stimulated GH according to each type of GH provocation test between **(A)** Obese *vs*. Normal/OW in IGHD, **(B)** total IGHD *vs*. total OGHD, **(C)** Obese *vs*. Normal/OW in total GHD. *GH* growth hormone, *GHD* growth hormone deficiency, *IGHD* idiopathic growth hormone deficiency, *OGHD* organic growth hormone deficiency, *OW* overweight.

Peak-stimulated GH levels for different types of stimulation tests are shown in Fig. 2. The peak GH was generally low in the Obese group compared to the Normal/OW group, with the exception of the results from the L-dopa stimulation test (Fig. 2A–C). Specifically, the peak GH via the clonidine stimulation test in the obese IGHD group (0.86) was much lower compared to the Normal/OW group (5.68) (*P* = 0.006; Fig. 2A). The peak GH of the IGHD group (6.61) was significantly higher compared to the OGHD group (2.98) (*P* < 0.001) (Fig. 2B). Peak-stimulated GH according to type of provocation test was highest with clonidine followed by insulin and L-dopa, among all three groups in IGHD and OGHD, but the difference was statistically significant (Fig. 2B, Supplementary Fig. S2). The median peak GH of CGHD was 3.63 and that of PGHD was 7.46, and there was no significant difference in peak GH according to obesity status in both the CGHD and PGHD groups (Table 3).

**Table 3:** Peak stimulated GH according to provocation test in CGHD and PGHD.

Peak GH	Complete GHD			Partial GHD			CGHD	PGHD	*P* value
	Obese (n = 9)	Normal/OW (n = 105)	*P* value	Obese (n = 17)	Normal/OW (n = 308)	*P* value	Total (n = 114)	Total (n = 325)	
Total	3.8 (1.78, 4.5)	3.6 (2.51, 4.47)	NS	8 (6.33, 8.9)	7.46 (6.33, 8.68)	NS	3.63 (2.5, 4.5)	7.46 (6.33, 8.75)	<0.0001
Insulin	3.8 (1.78, 4.5)	2.8 (1.68, 3.76)	NS	6.53 (4.7, 8.75)	6.21 (4.17, 7.97)	NS	2.88 (1.68, 3.8)	6.21 (4.25, 8.0)	<0.0001
Clonidine	0.59 (0.32, 0.86)	2.8 (1.78, 3.59)	NS	2.04 (2.04, 2.04)	6.27 (4.63, 8.07)	NS	2.69 (0.86, 3.59)	6.24 (4.59, 8.06)	<0.0001
L-dopa	2.64 (1.32, 3.8)	2.8 (1.84, 4.0)	NS	6.33 (5.24, 8.0)	5.77 (3.85, 7.3)	NS	2.8 (1.84, 4.0)	5.78 (3.91, 7.37)	<0.0001

Data are expressed as median (IQR).

^*^Significant association was classified as *P* < 0.05.

*CGHD* complete growth hormone deficiency, *GH* growth hormone, *GHD* growth hormone deficiency, *NS* not significant, *OW* overweight, *PGHD* partial growth hormone deficiency.

### Peak-stimulated GH and obesity

**Figure 3:**
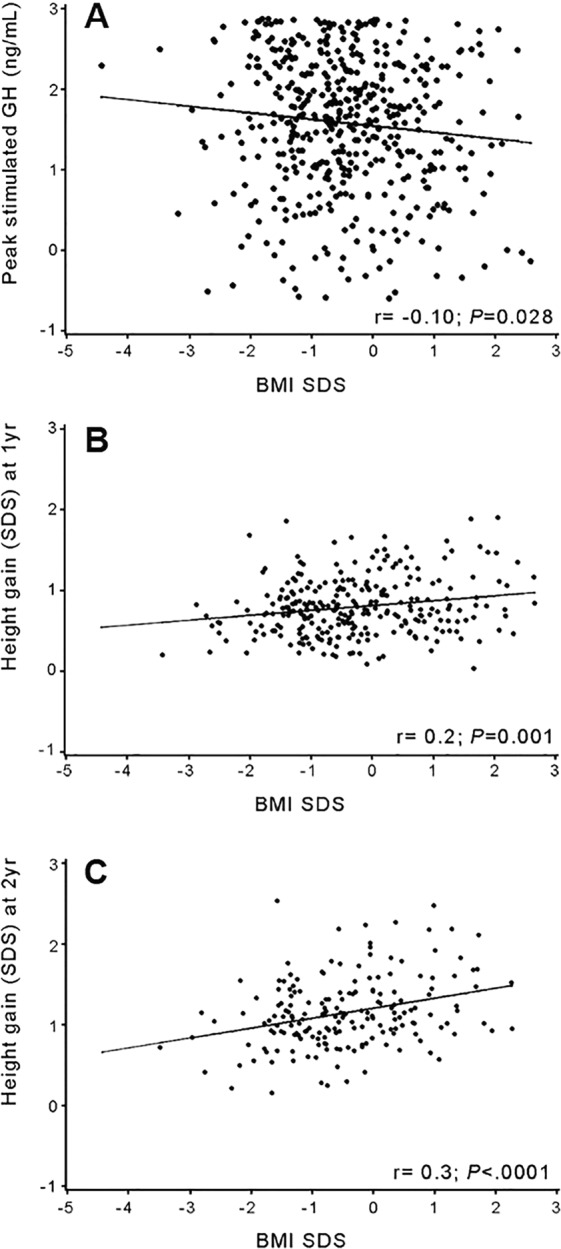
Univariate regression analyses demonstrating association between BMI SDS and **(A)** peak-stimulated GH, **(B)** height gain SDS at 1 year of GHT, and **(C)** height gain SDS at 2 years of GHT in IGHD subjects. *BMI* body mass index, *GHT* growth hormone treatment, *IGHD* idiopathic growth hormone deficiency, *SDS* standard deviation score.

The Pearson correlation coefficient test and stepwise multiple regression analysis which were used to identify the relationship between peak-stimulated GH and other related variables showed a negative correlation of peak GH decreasing by 1.06 for every 1.0 increase in BMI in the IGHD group (*P* = 0.026; Table 4). The cutoff point of peak-stimulated GH according to obesity was 5.57 in total GHD and IGHD, 8.75 in PGHD, 1.78 in CGHD, and 1.7 in OGHD (Supplementary Table S1). When comparing BMI SDS with a cutoff value of 5.57 for obesity in IGHD, if the peak-stimulated GH level was below 5.57, the mean BMI SDS was −0.13 SDS, significantly higher than the mean BMI of −0.37 SDS, when peak-stimulated GH was 5.57 and above (*P* = 0.03) (data not shown). The relationship between BMI SDS and GH peak in IGHD showed a very weak negative correlation despite achieving statistical significance (GHD *r* = −0.10, *P* = 0.028; Fig. 3A).

**Table 4:** Stepwise multiple regression analysis of peak-stimulated GH and other factors in IGHD.

	Regression Coefficient	Standard error	β	*P*-value
Intercept	3.92	0.33	0	<0.0001
BMI SDS	-1.06	0.39	-0.69	0.026

Exploratory variable was only BMI SDS.

*BMI* body mass index, *GH*, growth hormone, *IGHD* idiopathic growth hormone deficiency, *OW*, overweight; *SDS*, standard deviation score.

β: Standardized partial regression coefficient.

Adjusted R^2^ = 0.42.

^*^Significant association was classified as *P* < 0.05.

### Changes in height SDS and BMI SDS during GHT

**Figure 4:**
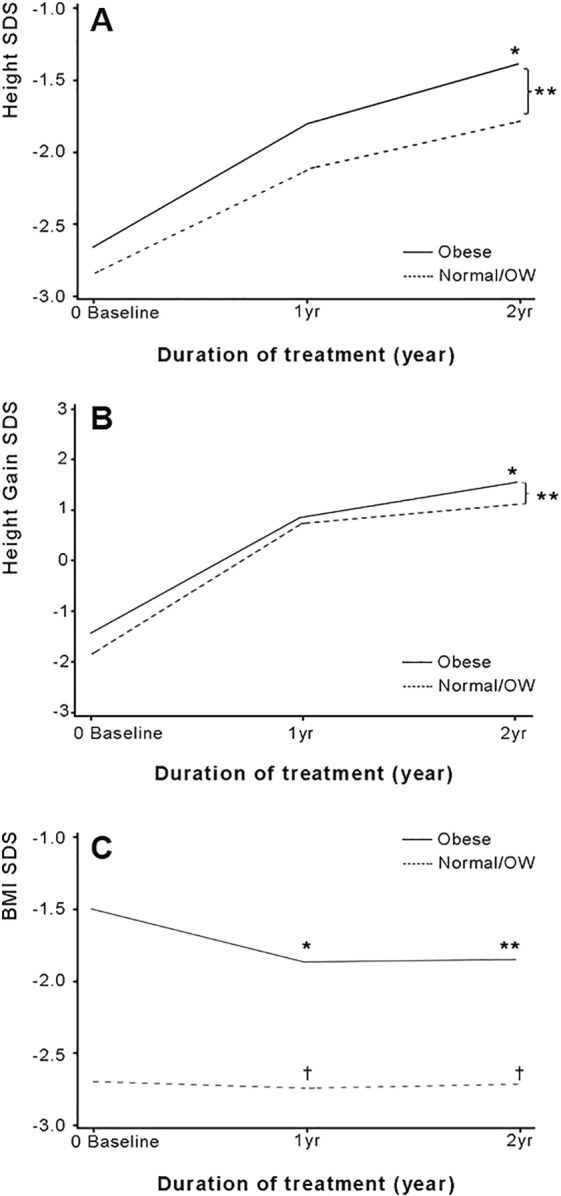
Height SDS, height gain SDS, and BMI SDS in the Obese and Normal/OW groups of IGHD from baseline to 2 years of GHT. *P* value was obtained from the Wilcoxon rank-sum test, two-sample *t*-test, or paired *t*-test. **(A)** Median height SDS at baseline, year 1, and year 2. ^*^P < 0.001 represents the difference in changes from baseline for each group of Obese and Normal/OW. ^**^P < 0.05 represents the difference in changes between the Obese and Normal/OW groups. **(B)** Median height gain SDS at year 1 and year 2. ^*^P < 0.001 represents the difference in changes from baseline for each group of Obese and Normal/OW. ^**^P = 0.03 represents the difference in changes between Obese and Normal/OW groups. **(C)** Median BMI SDS at baseline, year 1, and year 2. ^*^(*P* = 0.0001, at year 1) and ^**^(*P* = 0.004, at year 2) represent the difference in changes from baseline for obese group. The *P* value was not significant for the Normal/OW group (^†^not significant). *BMI* body mass index, *GHT* growth hormone treatment, *IGHD* idiopathic growth hormone deficiency, *OW* overweight, *SDS* standard deviation score.

In IGHD, the height SDS (Fig. 4A) showed the most significant increase in value of up to 2 years of GHT compared to baseline (^*^, *P* < 0.001) with a significant difference between the Obese and Normal/OW groups (^**^, *P* < 0.05). Height gain SDS (Fig. 4B) also showed a significant increase in 2 years with a marked increase during the first year of GHT (^*^, *P* < 0.001), but there was no significant difference between the Obese and Normal/OW groups for 1-year height gain SDS. Conversely, height gain SDS in the second year of GHT was significantly higher in the Obese group than in the Normal/OW group (^**^, *P* = 0.03). The BMI SDS (Fig. 4C) in the Obese group showed a significant decrease during 2 years of GHT (^**^, *P* = 0.0001) from baseline, while the Normal/OW group had no marked decline. In particular, the Obese group showed the largest decline for the first year of GHT (^*^, *P* = 0.004). Height gain SDS and BMI SDS showed a positive correlation over 2 years of GHT in IGHD (Fig. 3B [at 1 year, *r* = 0.2, *P* < 0.001]; Fig. 3C [at 2 years, *r* = 0.3, *P* < 0.0001]). There was no correlation between height gain SDS and BMI SDS in OGHD (*r* = −0.08, *P* = 0.77). When comparing the changes in height SDS for 2 years in IGHD, OGHD, CGHD, and PGHD (Supplementary Fig. S3), each group showed a significant height increase from baseline. In particular, the height SDS in the OGHD group for the second year of treatment was significantly higher compared to the IGHD group (*P* = 0.014), and there was no significant difference between the CGHD and PGHD groups. Moreover, OGHD group showed significantly decreased BMI compared to IGHD group in the second year of GHT (*P* = 0.015). There was no significant difference in the change of BMI SDS between the CGHD and PGHD groups during GHT (Supplementary Fig. S3).

## Discussion

This study investigated a large pediatric cohort of patients with GHD from the LG growth study who underwent a GHT and serial endocrinologic assessment during 2 years of follow-up. Our results demonstrated a significant relationship between peak-stimulated GH on the GH provocation test and obesity. There was a negative correlation between BMI SDS and peak-stimulated GH, suggesting that there was relatively less peak-stimulated GH in obese children, thereby highlighting the possibility of an increased false positive diagnosis of GHD.

In our study, peak-stimulated GH was not associated with serum IGF-1 level, but IGF-1 was higher and the therapeutic response to GHT was better in other groups compared to the obese group. However, data on serum IGF-I level in obese individuals are controversial, with studies reporting low^21–24^, high^25–27^, and normal levels^28,29^. Moreover, some studies have reported no relationship between IGF-1 and BMI SDS, and increased IGF-1 in obese GHD which supports our results^30^. This suggests that a modified GH-IGF-1 axis in obese children can only be meaningfully interpreted by measuring free IGF-1 to determine the true effects of BMI considering various factors^31–33^.

There have been few studies on the impact of BMI on GHT in children with GHD; the studies showed an inverse relationship between BMI and first-year growth response^34^, or vice versa^30^. Hawcutt *et al*.^30^ showed that height gain SDS was directly related to BMI SDS during the first year of treatment, except in obese patients. Our study results as presented in Fig. 4(A,B), showed a relatively higher increment of height SDS in the obese group over 2 years of treatment compared to the Normal/OW group. These results might be attributed not only to differences between study period and subjects, but also to differences in the definition of obesity. In particular, our study defined obesity as BMI equal to or greater than the 95th percentile for age and gender based on the updated Centers for Disease Control and Prevention guidelines^35^ compared to Hawcutt *et al*.^30^ who defined obesity as BMI SDS ≥ 2.0. However, Hawcutt *et al*. found a similar results to our study suggesting that gain in height and IGF-1 SDS has a positive relationship to BMI SDS, which might result from relative higher dose per weight (kg base) and higher IGF-1^36^. In addition, the 2-year upward curve of treatment slightly decreased from the first year of GHT due to the ‘ceiling effect’ despite a significant increase of height SDS in each group regardless of BMI. This result is noteworthy in that height gain SDS from the first to second year of GHT in the Obese group was significantly greater compared to the Normal/OW group, indicating that the effect of GHT was relatively well maintained even up to the second year of GHT in obese children with GHD. Cole *et al*.^37^ also found that the greatest growth response was in the first year of GHT which waned thereafter, and short obese children with low peak GH close to typical phenotype of GHD showed the best response to GHT, which is consistent with our results.

Another notable result was the significant decrease in BMI in the first year of GHT in the Obese group (Fig. 4C), which might be interpreted as showing a better response to GHT when closer to the classic clinical features of GHD. However, similar to the height gain results, there was no significant continuous decrease in BMI after the first year of treatment.

In contrast to the close relationship between the results of the GH provocation test and the growth effects of the first year of GHT shown in the study by Cole *et al*.^37^, there was no significant association between peak-stimulated GH and height gain SDS during the first year of GHT on multiple regression analysis regardless of BMI SDS in our study (Supplementary Table S2). Likewise, the results of the change of height SDS after 1 year of GHT showed no difference between the CGHD and PGHD groups (Supplementary Fig. S3). However, Cole *et al*.^37^ did not unify the pharmacologic stimuli for GH provocation test with one ITT requirement as done in our study, and their study involved smaller sample size. These differences in study design and the difference of classification by separating CGHD and PGHD within IGHD and not including OGHD may have resulted in different outcomes.

The degree of obesity in GHD children is strongly affected by the etiology of GHD^38,39^. Patients with OGHD (i.e., the GHD status caused by organic pituitary damage) have a higher BMI-SDS than patients with IGHD^18,40^, which is in agreement with our study results confirming the relatively high trend of BMI SDS and weight SDS of OGHD compared to IGHD. Our results also showed that peak GH in obese patients with GHD was significantly lower compared to patients with IGHD, demonstrating the negative correlation of BMI and peak GH. In addition, the results of significantly reduced peak stimulated GH (OGHD, 2.98 *vs*. IGHD, 6.61; Fig. 2B and significant height increment in OGHD compared to IGHD (Supplementary Fig. S3) are consistent with findings from previous studies^41–43^. That is, this is in line with prior studies suggesting the need for BMI-specific cutoff point for the diagnosis of GHD in patients with pituitary disease^13,15,44^.

The GH provocation test is a routine procedure for the diagnosis of GHD despite its poor diagnostic accuracy. The current diagnostic cut-off value for GHD is 10 mcg/L, but this value remains controversial given the effects of variable factors and possible different potencies of each pharmacologic stimuli on the GHST. The participants in our study had the essential inclusion conditions for ITT, followed by the L-dopa and clonidine stimulation test. The types of provocation tests with the highest peak-stimulated GH in total GHD, IGHD, and OGHD were the same in order of clonidine, insulin, and L-dopa, but did not show a significant difference. Ghigo *et al*.^45^ also found no difference in peak GH levels with different pharmacologic GH stimuli, with the exception of GHRH.

On the other hand, peak-stimulated GH in the obese GHD group diagnosed with the clonidine test showed significantly lower peak-stimulated GH compared to those who were not obese despite the lack of data (Peak GH: 0.86 [Obese] *vs*. 5.49 [Normal/OW], *P* = 0.008). The results of insulin, L-dopa, and clonidine-induced GH provocation tests in IGHD showed that the cutoff point for CGHD in the Obese group was significantly lower compared to the Normal/OW group. Given these results, obesity varies from type of provocation test, but has a common significant impact on peak-stimulated GH level. Loche *et al*.^19^ and Lee *et al*.^16^ also identified the negative impact of BMI on GH response to the clonidine stimulation test. While both studies compared only one or two GHSTs on ISS and not GHD patients, we additionally analysed three different stimuli, namely insulin, L-dopa, and clonidine. By comparing to the controls (“simple obesity” group), it seems necessary to set the cutoff value of GHST to a lower value in the obese GHD group including clonidine-induced tests.

There were several limitations in this study. First, this was a retrospective cross-sectional study, thus unable to establish causal relationship and is also prone to confounding bias. To overcome this limitation, we selected patients in a homogeneous group based on our own strict inclusion and exclusion criteria to exclude as much confounding bias as possible. Second, our patients did not receive sex-steroid priming before GHST, which might be advantageous for children in the transition period to reduce the number of false-positive tests. However, this is not a commonly recommended procedure for GHST because of the lack of consensus guidelines on the use of priming^46,47^. Moreover, our patients were all prepubertal, and did not need sex-steroid priming. Third, we did not have data on potential stronger correlates of GH secretion such as waist to hip ratio, serum lipid profile, and cardiovascular parameters. Nonetheless, recent studies have shown that BMI can function as a more specific measure of visceral abdominal obesity, a strong association of GH secretion^48,49^ and that it is strongly correlated with overall body fat, as such, waist circumference and BMI analysis alone are considered sufficiently meaningful. Lastly, only 12.8% of total study participants were overweight or obese, which makes it difficult to make strong claims about the role of obesity in GHT. Such limitation might result from a different definition of obesity from adults and the relatively lower prevalence of obesity in children. Further large-scale studies to compare obese GHD and simple obese children are required.

Our study also has some key strengths. First, this study was conducted in a large homogeneous sample size of prepubertal children with GHD. In addition, ITT was included as common provocative test and at least two or more stimulation tests were performed which improving the precision of the analysis. Second, various parameters were actively analysed among the patients who were classified as obese, overweight, and normal based on BMI. Moreover, we were able to obtain more diverse information by dividing the patients into IGHD, OGHD, CGHD, and PGHD and implementing subdivision and systematic classification and analysis. Although a few previous studies consisting of GHD patients exist^8,19,20^, there has been no study involving a large cohort of prepubertal GHD patients like ours. In addition, the relationship between peak GH and BMI has mostly been investigated in the heterogenous short stature group including ISS without grouping of BMI according to childhood obesity diagnostic criteria.

In conclusion, we identified a significant negative relationship between peak-stimulated GH level by the GH provocation test and obesity in prepubertal children with GHD and found that obesity was associated with the treatment effect of GH, although, there were differences in obesity levels among pharmacologic stimuli used for the GH provocation test. Our findings emphasise the need to reset a diagnostic cutoff value for children with GHD and to consider different provocation tests that take obesity into account. We hope that the results of this study draw attention towards the potential overdiagnosis of GHD in obese patients due to their negative impact on the GH provocation test. Larger studies that include obese children are needed to investigate the clinical application and establish causal relationships between BMI and peak GH.

## Methods

### Study design and patients

A total of 1297 patients with GHD from multiple centres were screened from December 2011 to March 2017 for this cross-sectional, observational study, which was registered as an LG Growth study. The study fully achieved the Declaration of Helsinki, and written informed consent was obtained from a parent or legal guardian for study participation. The study protocol was approved by the Ethics Committee of Inha University Hospital (2017-12-009-001). A schematic diagram of selection entry is shown in Fig. 1. The inclusion criteria were: (1) GHD defined as peak serum GH concentration <10 ng/mL upon provocation with a combination of at least two separate stimulation tests^3,50,51^ that includes the insulin stimulation test (ITT); (2) diagnosis with GHD prior to puberty (Tanner stage 1; testicular volume of 4 cc or less (boys) and Tanner stage I breast development [girls]); (3) height below the third percentile for age and sex (data from the 2017 Korean National Growth Charts for children and adolescents^52^) (for IGHD only); (4) chronological age (CA) between 3 and 11 years for boys and between 3 and 10 years for girls (5) Bone age (BA) of at least 6 months younger than CA upon diagnosis with GHD; (6) presence of baseline demographic data including height and weight; (7) follow-up period longer than 1 year after GHT; (8) initiation of GHT within 1 month after diagnosis with GHD; and (9) naïve to GHT. The exclusion criteria were: (1) chronic illness such as chronic kidney disease, malnutrition, and immunodeficiency; (2) overt diabetes mellitus; (3) chromosomal abnormalities and medical syndromes (e.g., Turner’s syndrome, Laron syndrome, SHOX gene deficiency, Prader-Willi syndrome, Noonan syndrome, Russell-Silver syndrome); (4) prematurity (gestational age <30 weeks), small for gestational age; and (5) other skeletal dysplasia.

A total of 460 patients met the inclusion criteria and were selected for the study. We divided the GHD patients into IGHD and OGHD (i.e., trauma, cranial irradiation, brain tumor or another organic pituitary abnormally) groups. The IGHD was divided again into CGHD and PGHD groups. We defined CGHD as peak GH < 5 μg/L in both provocation tests, and PGHD as peak GH ≥ 5–10 μg/L in more than one test at diagnosis as proposed in prior studies^53,54^. Each group was also divided into three groups as “Obese” (i.e., BMI ≥ 95th percentile for age and gender), “OW” (i.e., BMI percentile for age and gender from the 85th to 95th percentile), and “Normal” (i.e., BMI 5th to <85th percentile for age and gender)^55^.

### Endocrine studies

Patients who performed both baseline serum IGF-1 and IGFBP-3 and GHST were sampled the same day. The ITT was conducted, and the other GHST was performed using clonidine and Levodopa (L-dopa). L-dopa (body weight >30 kg, 500 mg; 15–30 kg, 250 mg; <15 kg, 125 mg; Sinemet®; MSD, Whitehouse Station, NJ, USA) and clonidine (0.125 mg/m^2^; Clonidine Hydrochloride®, Mylan Pharmaceuticals, Canonsburg, PA, USA) was orally administered after 8–10 h of overnight fasting without sex steroid priming. Blood samples were collected at 0, 30, 45, 60, 90, and 120 min. Regular insulin (0.1 IU/kg) was administered intravenously as a bolus at time 0 to stimulate hypoglycaemia (blood glucose <45 mg/dL or <one-half of baseline glucose level). Plasma samples for ITT were drawn at 0, 15, 30, 45, 60, 90, and 120 min, where time 0 is the initiation of insulin injection. BA was measured using the method described by Greulich and Pyle^56^. All height, weight, and BMI standard deviation score (SDS) were calculated using the 2017 Korean National Growth Charts^52^. Serum IGF-1 levels were measured by an immunoradiometric assay (Immunotech, Marseille Cedex, France). The sensitivity of the assay was 2 ng/mL. The intra- and inter-assay coefficients of variations were less than 6.3% and 6.8%, respectively.

### Statistical analysis

Statistical analyses were performed using SAS® version 9.4 (SAS Institute, Inc., Cary, NC, USA). Variables were expressed as the medians (interquartile ranges) for non-normally distributed variables. Comparisons between groups of categorical variables were performed using the Fisher’s exact test or chi-square test. Comparisons demographic data and peak-stimulated GH between groups were performed using the two sample *t*-test, Wilcoxon rank-sum test, analysis of variance, and Kruskal-Wallis Test. Differences from baseline for height SDS and BMI SDS during GHT were compared using the paired *t*-test or Wilcoxon signed-rank test. Bivariate associations between peak-stimulated GH and BMI were assessed with the Pearson correlation coefficient. Associated factors with peak-stimulated GH were analysed using stepwise multivariate analysis. Receiver operating characteristic (ROC) curve analysis was conducted for peak-stimulated GH according to obesity. The area below the curves was calculated with 95% confidence intervals. Significance was defined as *P* < 0.05.

## Supplementary information

Supplementaryinformation

## Data Availability

All data generated or analysed during this study are included in this published article (and its supplementary information files).
