# Supplementary material for: Impact of BMI on peak growth hormone responses to provocative tests and therapeutic outcome in children with growth hormone deficiency
Source: Sci Rep. 2019 Nov 7;9:16181. doi: 10.1038/s41598-019-52644-1 (PMC6838176; doi:10.1038/s41598-019-52644-1)
Supplement: Supplementary file 1 — Supplementaryinformation [file 41598_2019_52644_MOESM1_ESM.docx]

**Supplementary information**

**Impact of BMI on peak growth hormone responses to provocative tests and therapeutic outcome in children with growth hormone deficiency**

Aram Yang^1^, Sung Yoon Cho^2^, Min Jung Kwak^3^, Su Jin Kim^4^, Sung Won Park^5^, Dong-Kyu Jin^2^, Ji-Eun Lee^4,*^

**Table of Contents**

**Table S1**- Cut off points of peak growth hormone for obesity in total GHD, IGHD, OGHD, CGHD, and PGHD.

**Table S2**- Correlation between peak-stimulated GH in five groups classified by BMI SDS and 1-year height gain SDS in IGHD.

**Figure S1**- Change in IGF-1 SDS from baseline during GHT for the Obese and Normal/OW groups in IGHD.

**Figure S2**- Comparison of response of peak-stimulated GH among three groups (total GHD, IGHD, OGHD) according to the type of provocation test.

**Figure S3**- Changes in height SDS and BMI SDS after GHT from baseline in the IGHD, OGHD, CGHD, and PGHD groups.

**Supplementary Table S1.** Cut off points of peak growth hormone for obesity in total GHD, IGHD, OGHD, CGHD, and PGHD.

|  | **Idiopathic GHD** | **Organic GHD** | **Complete GHD** | **Partial GHD** | **Total GHD** |
| --- | --- | --- | --- | --- | --- |
| Cut off point | 5.57 | 1.7 | 1.78 | 8.75 | 5.57 |
| Sensitivity | 0.5 | 1.0 | 0.33 | 0.47 | 0.54 |
| Specificity | 0.68 | 0.68 | 0.87 | 0.76 | 0.66 |
| AUC (95% CI) | 0.54 (0.41, 0.66) | 0.71 (0.50, 0.92) | 0.52 (0.29, 0.74) | 0.52 (0.36, 0.68) | 0.56 (0.44, 0.68) |

*AUC* area under the ROC curve, *CI* confidence interval, *GHD* growth hormone deficiency.

**Supplementary Table S2.** Correlation between peak-stimulated GH in five groups classified by BMI SDS and 1-year height gain SDS in IGHD.

|  | | **Normal**  **(n=236)** | **Overweight**  **(n=19)** | **Obese**  **(n=20)** | **Normal/OW (n=272)** | **Total**  **(n=275)** |
| --- | --- | --- | --- | --- | --- | --- |
| 1year height gain SDS | Pearson correlation | -0.056 | 0.143 | -0.078 | -0.023 | -0.058 |
|  | *P*-value | 0.391 | 0.559 | 0.745 | 0.701 | 0.341 |
|  | N | 236 | 19 | 20 | 272 | 275 |

^*^Significant association was classified as *P*<0.05.

*SDS* standard deviation score.

**Supplementary Figure S1.** Change in IGF-1 SDS from baseline during GHT for the Obese and Normal/OW groups in IGHD.

**

**

Both the Obese and Normal/OW groups showed significant increases in IGF-1 SDS compared to baseline up to the first and second years of treatment (^*^, *P*<0.05). In particular, the IGF-1 level was significantly higher than that of the Normal/OW group in the first year of treatment for the Obese group (^**^, *P*<0.0001). There was no significant difference in IGF-1 SDS between the Obese and Normal/OW groups in the second year of GHT (^†^, not significant).

**Supplementary Figure S2.** Comparison of response of peak-stimulated GH among three groups (total GHD, IGHD, OGHD) according to the type of provocation test.





**Supplementary Figure S3.** Changes in height SDS and BMI SDS after GHT from baseline in the IGHD, OGHD, CGHD, and PGHD groups.

**
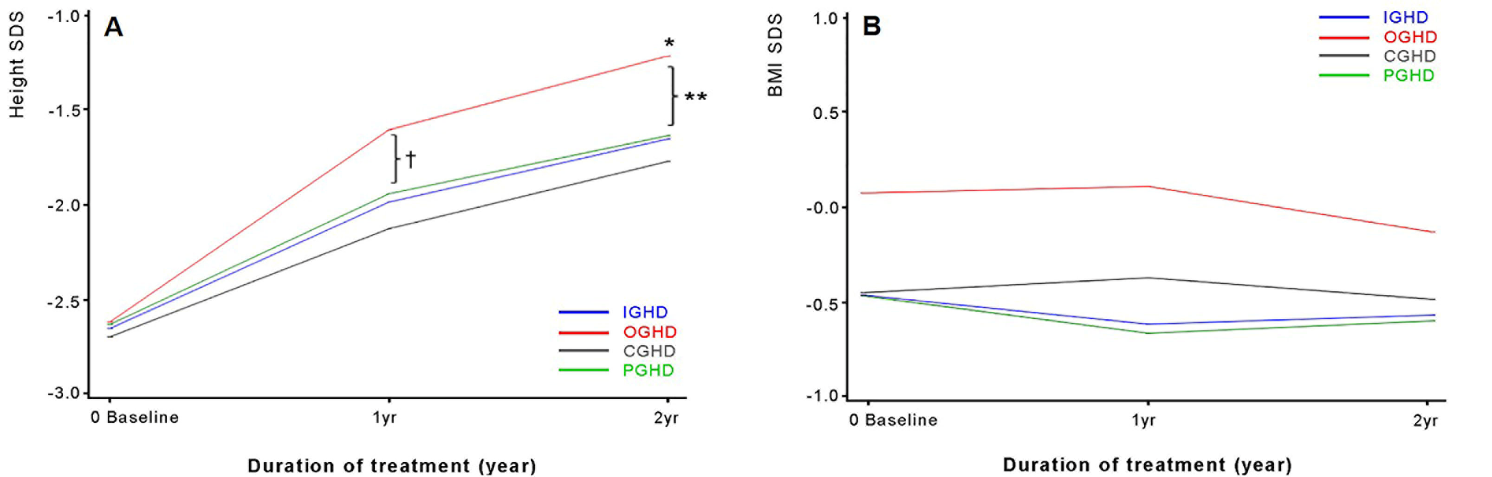
**

**(A)** All four groups (IGHD, OGHD, CGHD, PGHD) showed significant increases in height SDS up to the second year of treatment compared to the baseline (^*^, *P*<0.001). The difference in height SDS between IGHD and OGHD was not significant in the first year of treatment, but the second year was significant (^**^, *P*=0.014; ^†^, not significant).

**(B)** While the BMI SDS in IGHD and PGHD showed significant decreases up to the second year of GHT (*P*<0.05), OGHD and CGHD did not show significant decreases. The difference in BMI SDS between IGHD and OGHD was not significant in the first year of treatment, but the second year was significant (*P*=0.015). There was no significant change in BMI SDS between the CGHD and PGHD groups.
